# Cells and Stripes: A novel quantitative photo-manipulation technique

**DOI:** 10.1038/srep19567

**Published:** 2016-01-18

**Authors:** Martin Mistrik, Eva Vesela, Tomas Furst, Hana Hanzlikova, Ivo Frydrych, Jan Gursky, Dusana Majera, Jiri Bartek

**Affiliations:** 1 Institute of Molecular and Translational Medicine, Faculty of Medicine and Dentistry, Palacky University, Olomouc, Czech Republic; 2 Institute of Molecular Genetics of the ASCR, v. v. i., Prague, Czech Republic; 3 Danish Cancer Society Research Center, Copenhagen, Denmark

## Abstract

Laser micro-irradiation is a technology widely used in the DNA damage response, checkpoint signaling, chromatin remodeling and related research fields, to assess chromatin modifications and recruitment of diverse DNA damage sensors, mediators and repair proteins to sites of DNA lesions. While this approach has aided numerous discoveries related to cell biology, maintenance of genome integrity, aging and cancer, it has so far been limited by a tedious manual definition of laser-irradiated subcellular regions, with the ensuing restriction to only a small number of cells treated and analyzed in a single experiment. Here, we present an improved and versatile alternative to the micro-irradiation approach: Quantitative analysis of photo-manipulated samples using innovative settings of standard laser-scanning microscopes. Up to 200 cells are simultaneously exposed to a laser beam in a defined pattern of collinear rays. The induced striation pattern is then automatically evaluated by a simple algorithm, which provides a quantitative assessment of various laser-induced phenotypes in live or fixed cells. Overall, this new approach represents a more robust alternative to existing techniques, and provides a versatile tool for a wide range of applications in biomedicine.

Confocal laser scanning microscopy (LSM) enables precise spatio-temporal photo-manipulations based on LSM-embedded lasers in defined regions of interest (ROIs). These techniques include induction of DNA damage in cell nucleus^1^,^2^ and various photo-bleaching/activating procedures such as fluorescence recovery after photobleaching (FRAP)^3^.

Among the research areas that exploit these techniques, live-cell monitoring of factors involved in cellular responses to DNA damage has been particularly prominent. Compared to standard conventional methods commonly employed in the DNA damage field, laser-induced DNA damage provides multiple advantages, such as the ability to damage DNA in a certain time-point and in only part of the nucleus while the surrounding DNA stays intact, a feature especially attractive for dynamic analyses of protein recruitment^4^,^5^,^6^,^7^,^8^. Arguably the most deleterious lesions formed after laser micro-irradiation are DNA double strand breaks (DSB), a response to which involves a cascade of carefully orchestrated events, eventually leading to recruitment of factors participating in repair of DSBs via either homologous recombination or nonhmologous end joining pathways^9^. Among the main factors involved in the stepwise recruitment and assembly of the DSB response proteins is MDC1, a scaffold protein^10^,^11^ the GFP-tagged version of which was used as one of the DNA damage reporters in live-cell imaging experiments performed as part of our present study. MDC1 is one of the early-response proteins that forms a platform for recruitment of other important DDR factors (eg. RNF8, 53BP1, BRCA1, etc)^12^,^13^,^14^. The other protein we used as a GFP-tagged reporter is 53BP1, a protein that plays an important role in DSB repair pathway choice^15^. Finally, as a representative of factors involved in DNA repair of non-DSB lesions, we also employed FANCD2, a protein that becomes activated by monoubiquitination and plays a central role in the so-called Fanconi anemia (FA) pathway implicated in repair of DNA interstrand crosslinks, translesion DNA synthesis, nucleolytic incision and homologous recombination^16^.

In general, current approaches to the photo-manipulation techniques involve manual definition of the ROI, which is then exposed to a particular laser resulting in the respective photo-effect. The ROI is followed over time and its signal intensity is measured. The process is laborious and commonly requires constant attention for active repositioning of the readout area due to movement of cells over time^3^.

Here we propose a method to circumvent limitations of the manual approach by simultaneously exposing many cells to a co-linear pattern of laser rays. This is achieved by a particular set of LSM settings likely universally compatible with microscopes of various producers (tested on two types of LSM systems so far).

## Results

### Standard LSM systems can be adapted for photo-manipulation in a predefined striation pattern covering the entire acquisition area

**Figure 1:**
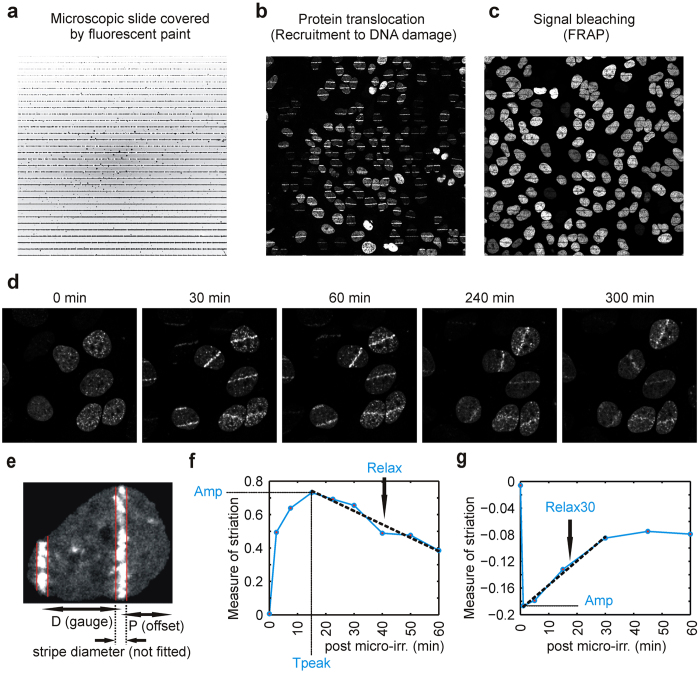
Method introduction. (**a**) Visualization of the striation pattern on a layer of fluorescent paint. (**b**) Striation pattern induced by a 355 nm laser (32 lines/field) in BrdU pre-sensitized U-2-OS-MDC1-GFP cells. DNA damage is visualized by recruitment of ectopically expressed MDC1-GFP protein. (**c**) Striation pattern bleached by a 488 nm laser (32 lines/field) in U-2-OS cells ectopically expressing histone H2B-GFP. (**d**) Evolution of the striation pattern over time. Cell line and DNA damage induction were the same as in (**b**). (**e**) Automatic stripe recognition by the software in the nucleus based on known values (gauge and position of the stripe) and additional fitting of recognized stripes. (**f**) Typical evolution of striation pattern after DNA damage caused by a 355 nm laser irradiation (32 lines/field, 1 iteration) in BrdU pre-sensitized U-2-OS-MDC1-GFP cells. The curve is plotted as medians of measure of striation (MS) values at indicated time points. The following parameters are used to describe the curve: Amp (amplitude, the maximum of MS over all time points), Tpeak (time to reach maximum MS), Relax (slope of the line fitted to MS values after Tpeak). (**g**) Typical evolution of striation pattern after bleaching. FRAP curve is formed by median values of MS at each time-point. The following parameters are used to describe the curve: Amp (amplitude, the minimum of MS), Relax30 (slope of the line fitted to MS values between 1 min and 30 min). Used cell line is the same as in (**c**).

Once the LSM system is set to a very low resolution and slow scanning speed, it generates a specific striation pattern. In principle, the entire acquisition area is scanned with small resolution e.g. only 32 pixels in each dimension (x and y). Laser scanning microscope does not switch off the lasers during the bi-directional scanning process between points nor lines. Such settings then result into a pattern of 32 horizontal lines, rather than 32 × 32 dots. The total dose of light energy for each of the lines can be adjusted by altering the scanning speed (pixel dwell time) and further by repetitions of the scanning cycles (iterations). This effect can be visualized for example on a microscopic slide covered with a thin layer of fluorescent paint which is bleached by the laser only within the scanning lines (Fig. 1a). If the used laser wavelength and power has DNA-damaging properties, the same spatially defined DNA damage striation pattern can be induced in live cells and visualized for example by translocation of tagged responsive proteins towards the DNA lesions (Fig. 1b). If the laser is set to a bleaching mode, a similar negative striation pattern is achieved (Fig. 1c). A large number of cells (limited only by the numbers of cells that can grow within the acquisition area) can be exposed in a very short time and the induced striation pattern of known parameters is amenable to an automatic software evaluation.

In the LSM-based laser-induced DNA damage applications, the amount of recruited and/or modified responsive proteins (DNA damage recognition) and their subsequent release, degradation or de-modification (processes reflecting DNA repair) can be measured. This is applicable to immunofluorescence (IF)^7^,^17^ as well as live-cell imaging^8^. IF enables simultaneous detection of multiple proteins, including protein modifications (Supplementary Fig. S1), but has the disadvantages of an end-point assay. Live-cell analysis based on reporter cell lines tracks individual cells over time. The evolution of the striation pattern is followed providing a unique readout for various dynamic studies^8^ (Fig. 1d).

### Principles of the signal quantification

The induced striation pattern is described by two pre-defined parameters: the stripe width and the inter-stripe distance (gauge). The stripe width is defined by the used hardware and cannot be manipulated by the user. On the other hand, the inter-stripe distance can be manipulated by changing the scanning resolution or objective magnification defining the total number of stripes per field (Supplementary Fig. S2). Simple software was developed for quantitative evaluation of the induced striation pattern. The algorithm first recognizes individual nuclei. In each nucleus, stripe(s) are automatically identified (see Fig. 1e and Methods for details and Supplementary information for the software code). A measure of striation (MS) is proposed to capture the excess signal in the stripes for each nucleus. Such an approach for DDR quantification was directly compared to a commonly used method measuring mean fluorescence signal per nucleus^18^. For fixed samples involving protein modifications at sites of DNA damage such as lesion-associated phosphorylation of histone H2AX, the MS provides better discrimination between positive and negative controls (Supplementary Fig. S1). For assessment of reporter protein recruitment, such as for MDC1-GFP accumulation at sites of DNA damage, only the MS value enables meaningful analysis of the DDR (Supplementary Fig. S1).

### Method optimization

In live-cell reporters involving GFP-tagged DNA-damage responsive proteins, the dependence of MS on time can be captured by plotting the median MS for each time-point. Such dependence can be quantified by the following three parameters: amplitude, time to peak response, and relaxation speed (Fig. 1f,g, see Methods for details). These parameters reflect changes in the amount of DNA damage and subsequent processes such as lesion recognition and repair. If DNA damage/repair processes are studied in time, the response curve should be optimized for each reporter cell line by proper settings of the striping system to avoid saturation. In case of LSM systems, the total laser irradiation dose is affected by multiple parameters such as the laser wavelength and power, the pre-sensitization strategy (Supplementary Fig. S3) and hardware settings including the light path, scanning speed and used objectives (Supplementary Fig. S2). Despite not absolutely necessary^19^ (see also Supplementary Fig. S3), BrdU pre-sensitization of cells is the ‘gold standard’ for damaging DNA by UV-A lasers^5^,^6^,^20^. The concentration of BrdU is one of the critical parameters affecting the induced damage^21^. Various BrdU concentrations were tested with the laser power fixed to cause minimal damage in non-presensitized cells (Supplementary Fig. S3). We found that the commonly used concentration range 1–10 μM of BrdU for 24 h is too high for quantitative readouts since levels above 1 μM cause saturation of the signal amplitude in various tested reporter cell lines and for both tested LSM systems (Supplementary Fig. S3). Moreover, concentrations above 1 μM cause unwanted interference with cell cycle progression (Supplementary Fig. S3). Thus, for further experiments, 24-hour pre-incubation with 0.5 μM BrdU was chosen as an optimum due to its minimal adverse effects and MS values below saturation.

### Method validation for DNA damage analysis

**Figure 2:**
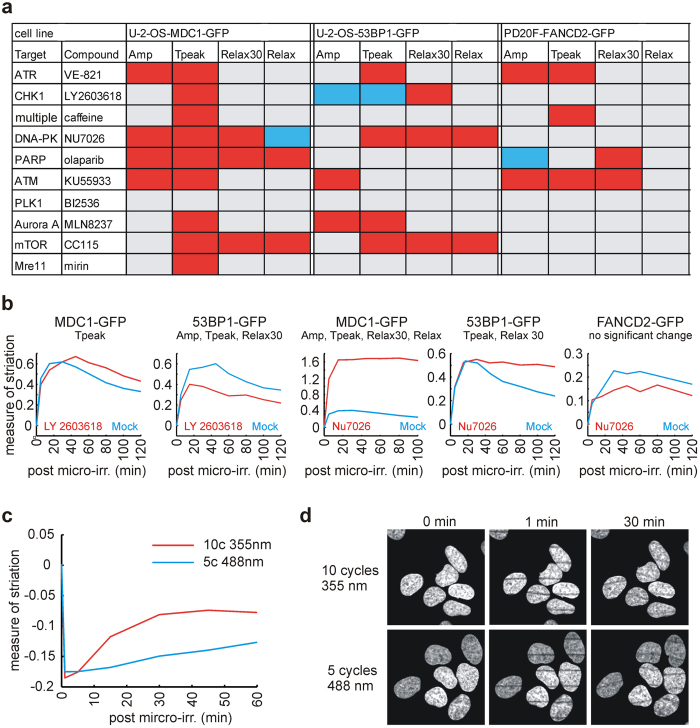
Method application. (**a**) Summary table for three reporter cell lines addressing parameters of DDR curve, Amp (amplitude, the maximum of MS over all time points), Tpeak (time to reach maximum MS), Relax (slope of the line fitted to DDR curve after Tpeak), Relax30 (slope of the line fitted to DDR curve after 30 min). The effects are color-coded: red – parameter upregulated, blue – downregulated, grey – no significant effect, Kruskal-Wallis test, p < 0.05. (**b**) Representative graphs of MS median values for selected compounds are showing differential reporter-dependent response (the parameters with significant change are listed in the graph titles), Kruskal-Wallis test, p < 0.05. (**c**) Illustrative graph of MS median values for H2B-GFP recovery after bleaching (FRAP). Bleaching was performed either with a 355 nm or 488 nm laser (32 lines/field) to the same level, Kruskal-Wallis test (difference in amplitude is non-significant, p = 0.87). Relax30 shows significant change (p < 1.E-4). (**d**) Illustrative images of U-2-OS-H2B-GFP cells bleached by a 355 nm or 488 nm laser and evolution of the striation pattern over time.

Once properly set, the striping method together with the software solution were tested on a small panel of known chemicals to prove its potential utility for applications involving large numbers of cells such as high-content screens for compounds and/or factors relevant for DNA integrity maintenance (potential cancer therapeutics). The compounds we selected are known to interfere with processes contributing to genomic stability, DNA damage signaling and/or repair pathways. The experiment was performed using a 96-well plate format in a semi-automatic regimen with autofocus and automatic repositioning and acquisition of striped regions in three different reporter cell lines. The tested compounds were added 2 hours before the experiment. For each compound, the cellular response at multiple time points after laser irradiation was compared with the mock treated sample. Four numerical parameters describing the MS curve (see Methods for details) were statistically evaluated (Fig. 2a). Notably, some of the compounds scored differentially for the MDC1-GFP, 53BP1-GFP and FANCD2-GFP reporters, respectively (Fig. 2a,b). Among the tested compounds only one (a PLK1 inhibitor) did not score in any of the measured parameters, in any of the reporter cell lines.

### Method validation for FRAP analysis

The presented method including the software solution is easily adaptable for other photo-manipulation techniques. As an example of its utility, the FRAP method was tested in U-2-OS H2B-GFP reporter cells. The cells were pre-sensitized by 0.5 μM BrdU for 24 h and bleached by the co-linear stripes with either 488 nm laser or 355 nm laser. MS evaluation was performed along the same principle as for live-cell analysis after DNA damage. Despite the signal was initially bleached to the same level for both lasers, the H2B-GFP signal recovery was significantly faster after the UV-A laser bleaching (Fig. 2c,d). Apart from their technical merit, these results with the UV-A laser induced damage are novel and consistent with a published report that DNA repair of lesions induced by UV-C lasers accelerates turnover of histones including H2B^22^, suggesting that accelerated histone turnover is a common feature shared by multiple DNA repair pathways.

## Discussion

The method presented in this study provides several advantages over currently used techniques, and offers a broad applicability in the area of LSM-based photo-manipulations in biomedicine. For the first time, a precisely defined striation pattern was used for simultaneous micro-irradiation of dozens of cells. Known parameters of striation patterns simplify software-based quantification of cellular responses. This enables an easy and unbiased evaluation of numbers of cells sufficient for robust statistical testing. Moreover, the method we present meets all requirements for full automation and application in high-content screens for new drugs and/or factors modulating diverse aspects of the DNA damage response. Several compounds that positively scored in our test panel are being intensively studied as potential anti-cancer therapeutics. Finally, the LSM system can produce the desired striation pattern rapidly because the laser path is ideally coordinated with the LSM scanning principle. This is particularly important for experiments where large numbers of cells are exposed simultaneously. Fast exposition speed allows also DNA damage experiments in non-pre-sensitized cells. Our data show that for DNA damage induction corresponding to pre-sensitization with 0.5 μM BrdU, the non-presensitized cells require approx. 6 times higher laser irradiation dose for the 355 nm, and a 10 times higher dose for the 405 nm laser (Supplementary Fig. S3). Thus either laser power or exposure time introduce serious limiting factors for presensitizer-free experiments if performed in a standard manner of manual laser path definition. Given all the advantages of the method we report here, we believe this new approach utilizing standard, widely used equipment could facilitate a broad range of biological and biomedical applications.

## Methods

### Microscopic devices

Microscopic station Zeiss Axioimager Z.1 with laser scanning LSM780 module. Lasers: UV-A 355 nm 65 mW (used for DNA damage induction, bleaching experiments and acquisition of Hoechst signal), argon-neon 458, 488, and 514 nm (used for acquisition of GFP and Alexa Flour 488 signals) and 568 nm (used for acquisition of Alexa Flour 568 signal). 40× objective (C-Apo, 1.2 DICIII, water immersion) was used for all experimental procedures unless stated otherwise in figure description. All other tested objectives are listed in Supplementary Fig. S2. Microscopic station Leica DMI 6000B with laser scanning TCS SP5 AOBS TANDEM module. Lasers: UV-A 405 nm 100 mW (used for DNA damage induction), argon-ion 458, 488, and 514 nm (used for acquisition of GFP signal). Objective: 40× objective (HC PL APO 40×/1.30 OIL CS2, LP/0,17/D). Both microscopic stations were equipped with temperature-controlled incubator for live-cell support.

### LSM settings for stripping procedure

ZEN 2011 software was used to control the Zeiss LSM780 device. For micro-irradiation 355 nm laser at maximum power (65 mW, 100% output) was used. To obtained desired striation pattern scan mode of very low resolution (size i.e. resolution 32 × 32, 64 × 64 or 128 × 128 pixels) and the lowest possible scanning speed (pixel dwell time 709.27 μs for 32 × 32 pixels) was used. Striping at pixel resolution 32 × 32 was chosen for most experiments with the 40× objective as it is causing approx. 1–2 stripes per cell nucleus. The scan was bidirectional, zoom 0.6. Number of iterations (means scanning cycles) is indicated in the figure legends (experiment dependent). Autofocus before each micro-irradiation was performed with 488 nm laser (the same settings as for respective GFP signal acquisition). For image acquisition in live-cell experiments with GFP-tagged reporters 488 nm laser was used. Images were acquired in 16 bit depth, zoom 0.6, five z-stack planes (0.6 μm apart). To minimize bleaching the autofocus for image acquisition was based on reflected light of 561 nm laser which allows visualization of a thin contact layer between cells and the glass cultivation surface (also known as backscatter image). For bleaching experiments either 488 nm laser was used (100% output), or 355 nm laser (100% output). Autofocus was performed in the same manner as for micro-irradiation experiments.

For live-cell experiments performed on Leica SP5, Leica Application Suite Advanced fluorescence, FRET, FRAP, Live Data software was used. Micro-irradiation was done with acquisition resolution 32 × 32 pixels, 10 Hz, bidirectional scan, 405 nm laser (68% output). Number of iterations (scanning cycles) is indicated in the figures. Acquisition of GFP signal was performed with following settings: resolution was set to 2048 × 2048, 70 Hz, 488 nm laser, Hyd5 detector, 12 bit image depth, no z-stack. Autofocus was performed on GFP signal with the same settings as acquisition.

All live cell experiments were performed in pre-heated incubator (37 °C). Plates were placed into the incubator at least 45 min before the experiment to ensure proper temperature equilibration.

### Visualization of the striation pattern using microscopic slide covered by fluorescent paint

Standard microscopic glass was covered by a homogeneous paint layer using red permanent marker (type: Permanent 8566, ink color 04, Centropen) and air dried. The paint layer was mounted by water and covered by a standard cover glass (0.17 mm). Signal was visualized using 561 nm laser and emission spectrum 570–710 nm. Micro-irradiation (stripping) procedure included 355 nm laser, 100%, 32 × 32 pixels, 1 iteration, pixel dwell time 709.27 μs, bi-directional scan.

### Cell lines and treatments

All reporter cell lines were stably expressing the respective protein with a GFP tag. U-2-OS-MDC1-GFP, U-2-OS-53BP1-GFP and PD20F-FANCD2-GFP were obtained from Danish Cancer Society Research Centre, Copenhagen, Denmark^8^,^23^, MRC-5 cells were obtained from ATCC and McCoy mouse fibroblasts were obtained from R-D Biotech. All cell lines were incubated under standard cultivation conditions (5% CO_2_, 37 °C), in DMEM (Gibco) supplemented with 10% FBS (Invitrogen). Cells were pretreated by 0.5 μM BrdU (Sigma) for 24 h before micro-irradiation unless stated otherwise (see figures description). For micro-irradiation procedures cells were seeded into 96 well plates with glass bottom (Corning) 18 hours before laser irradiation. 2 hours before laser irradiation the medium was changed to DMEM CO_2_ - independent medium without phenol red (Gibco) supplemented with 10% FBS and the tested chemicals. Cells were seeded in amounts to ensure sub-confluent density (proper cell cycle progression enables BrdU incorporation). After seeding the cells into 96 well plates the specimens were first placed on equilibrated bench for 20 min at RT to ensure equal cells distribution and then placed into incubator.

### U-2-OS-H2B-GFP clone construction

Commercial plasmid pBOS-H2BGFP Vector was used. Cells were electroporated with Neon Transfection System (Life Technologies) with 15 μg of plasmid per 1.10^6^ cells (pulse voltage - 1230 V, pulse width 10 ms, pulse number 2). 72 h after transfection, GFP-positive cells were sorted for bulk culture stably expressing H2B-GFP. After proper cell culture propagation, 1 cell per well sorting was done to obtain individual clones (BD FACS Aria).

### Quantitative analysis of the striation patterns

An in-house specialized software routine for the evaluation of the striation patterns was developed and implemented in MatLab. The first step of the routine consists of the segmentation of the images to find the individual nuclei. To be selected, a nucleus must satisfy certain conditions on its size and solidity. The process begins by segmenting the first image, acquired at the beginning of the experiment (*t* = 0, before laser irradiation). Standard thresholding algorithms (e.g. the Otsu’s method) do not perform well here because there is a large variance in the signal intensity among the individual nuclei (a common problem in most live-cells based reporter systems). Consequently, a cascade of decreasing local thresholds is used. After the cascade has finished, usually all the nuclei of desirable characteristics are identified in the image. For the segmentation of the subsequent images, a tracking algorithm is used. The algorithm attempts to track each nucleus by searching the vicinity of its positions in the previous image. This tracking approach is necessary because the signal intensity in the inter-stripe regions of the nuclei may decrease all the way to the background level and thus become completely “invisible” for any simple thresholding procedure.

Several measures were proposed and tested to quantify the evolution of the striation patterns induced by the laser within each recognized and over the time tracked nucleus. In the end, the relative excess signal in the stripes, hereafter called the measure of striation and abbreviated “MS”, proved to be the most useful. It is constructed as follows: Let us consider an image of an individual cell nucleus at time *t.* We start by rotating the image so that the (possible) stripes become vertical. This is done by taking the Radon Transform of the image and finding its maximum. If there are no stripes in the image, the nucleus gets usually rotated by a random angle.

Next, the position of the stripes and their diameter is found. This is done by fitting the stripes, i.e. maximizing a function of two variables *F*(*D,P*) where *D* stands for the gauge (i.e. the distance between the centers of the stripes) and *P* stands for the horizontal offset of the stripes (i.e. the position of the center of the first stripe from the rightmost boundary point of the nucleus, see Fig. 1e). The function *F* measures the ratio of the mean intensity of the signal in the stripes to the mean intensity of the signal in the entire nucleus. The width of the stripes is a user-defined constant and it is not fitted. Although both the parameters *D* and *P* are known from the initial setting of the laser, the optimization (i.e. fitting) step is necessary due to elasticity of the nuclei and their movement in time. Without this optimization step, the measure of striation would automatically decrease in time as the nuclei move around and change shape. This would create a systematic error – a false decrease in the intensity of the stripes. The minimization is implemented by means of a trivial algorithm that fully evaluates all plausible values of *D* and *P* and selects the highest value for *F*. The parameter *D* is allowed to take only integer values from a pre-specified range and the parameter *P* is not allowed to depart too much from the point where the vertical sum of the signal reaches its maximum. More advanced optimization procedures may naturally be used, however, this optimization step takes much less time than the cell-tracking part of the algorithm. Moreover, the full evaluation of a single image (which may contain around 100 nuclei) takes only few seconds on a standard PC.

Once the position of the stripes is found, the measure of striation is computed according to equation (1):


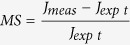


**Equation (1)**: *J*_*meas*_ (measured) stands for the actual signal integrated density of regions inside the stripes, and *J*_*expt*_ (expected) stands for the expected value of the signal integrated density in region of the stripes, if the nucleus was perfectly homogeneous and no stripes were present.

*J*_*expt*_ is computed according to equation (2):


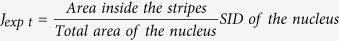


**Equation (2)**: SID stands for signal integrated density.

The measure *MS* thus captures the relative excess signal in the stripes. The baseline is zero which means there is no excess signal in the stripes, i.e. the stripes contain only as much signal as corresponds to their area, i.e. the signal is homogeneously distributed throughout the nucleus, i.e. there are no visible stripes. As the fluorescently labeled DNA-damage responsive proteins get recruited to the places of laser-induced damage (i.e. to the stripes), the measure of striation grows. After some time, the proteins are released, they begin to spread out again over the entire nucleus, and/or undergo degradation and the measure of striation decreases again. Thus, the measure of striation captures the dynamics of the DNA-repair processes. The construction of MS ensured that it is independent of the total SID of the nucleus. This is necessary because the total SID may vary greatly among individual nuclei.

### DNA damage repair (DDR) curve parameters

By a DDR curve we mean the dependence of MS on time for a particular nucleus. To compare different DDR curves, the following three parameters were defined (Fig. 1f,g):

1. The amplitude of the response (Amp) which measures the maximum amount of the protein recruited to the stripes. It is computed as the maximum of MS over all observed time points.
2. The time to peak response (Tpeak) measures the speed of the protein recruitment. It is computed as the time (in minutes) from the micro-irradiation until the MS reaches its maximum.
3. The relaxation speed (Relax) which reflects the dynamics of the repair process after the peak response. It is calculated as the slope of the line fitted to the function *MS*(*t*) for *t* > *Tpeak*, where *MS* stands for the measure of striation, and *t* for time. For some samples treated by DNA damage/repair interfering compounds, the MS did not peak during the observed period and kept increasing. In these cases, the relaxation speed was measured by the slope of the line fitted to *MS*(*t*) for *t* > *30* *min*).

These parameters are computed for every nucleus within a sample and differences among samples are tested for statistical significance by means of the Kruskal-Wallis test. The significance level of all tests was set to 0.05. For illustrative graphs the median MS at each timepoint was computed and these medians were connected by lines (Figs 1f,g and 2b).

### Screen for compounds potentially affecting DNA damage response

Panel of compounds including VE-821^24^ (Selleck Chem.), LY2603618^25^ (Selleck Chem.), Caffeine^26^ (Sigma), NU7026^27^ (Sigma), Olaparib^28^ (Selleck Chem.), KU55933^29^ (Sigma), BI2536^30^ (Selleck Chem.), MLN8237^31^ (Selleck Chem.), CC115^32^ (Celgene Corporation) and Mirin^33^ (Sigma) was tested in 3 reporter cell lines. Each compound was added to cells 2 hours before micro-irradiation in final concentration of 10 μM with the exception of BI2563 (100 nM). Respective DDR protein recruitment to sites of damage was evaluated as MS in each timepoint for every single cell. Four parameters characterizing the DDR curve (see above) were calculated and statistically tested against mock treated control (Fig. 2a). Every sample was tested in technical duplicate (i.e. in two separate wells on the same 96 well plate). Results from duplicates were pooled and tested against pooled mock treated sample. At least two independent biological replicates were performed for each tested compound. Only significant effects scored similarly in both biological replicates were marked in the table by a color change (Fig. 2a). Minimum of 100 cells were scored in each experiment.

### FRAP experiment

Cells were seeded and treated in the same way as for the micro-irradiation experiments, including BrdU pre-sensitization. The FRAP curve (i.e. the evolution of MS in time) was constructed in the same way as the DDR curve. The first two DDR curve parameters (Amp, Tpeak) were defined similarly as above (taking the minimum of MS instead of a maximum). Tpeak was not used as the minimum MS (i.e. maximum striation) is always reached immediately after the bleaching. The relaxation speed was measured in a slightly different way. The parameter Relax30 denotes the slope of the line fitted to the FRAP curve between t = 1 min and t = 30 min, the parameter Relax denotes the slope of the line fitted to the FRAP curve between t = 1 min and t = 60 min (Fig. 1g). Experiment was performed in two biological replicates. Minimum of 150 cells were scored in each experiment.

### Immunofluorescence

Cells were seeded and treated with BrdU in the same way as described for the live-cell experiments (see above). At indicated timepoints after micro-irradiation, cells were fixed with 10% buffered formalin (Sigma) and permeabilized with 0.5% Triton X for 5 min. After 20 min blocking in 1% BSA in PBS, the samples were incubated with primary antibodies at 4 °C 18 h, followed by 1 h incubation with secondary antibodies at RT. DNA was stained by Hoechst33342 (Invitrogen) 5 μg/ml in PBS. Acquisition of IF labeled samples was performed by the Zeiss LSM780 system. Scan mode was set to frame, size 1024 × 1024, 16 bit image depth, zoom 0.6, five z-stack planes (0.6 μm apart). Autofocus was performed with settings used for acquisition of Hoechst stained nuclei. Acquired images were analyzed for measure of striation by slightly modified software routine described above. The modification included deactivation of tracking module and recognition of individual nuclei based on Hoechst stain channel. Following antibodies were used: phospho-H2AX (Ser139) JBW301 (Millipore, 500×), 53BP1 (Santa Cruz, 500×), BRCA1 (Santa Cruz, 300×), cyclin A (Leica, 50×), AlexaFluor488 and AlexaFluor568 (Invitrogen, 1000×).

### Cell cycle analysis

Cells were seeded and treated in the same way as for the micro-irradiation experiment. After 24 h incubation with indicated concentrations of BrdU, cells were fixed with 10% formalin (Sigma) and stained with Hoechst33342 5 μg/ml in PBS (Invitrogen) for 10 min. Cell cycle was evaluated using OlympusBX71 inverted microscope and ScanR Acquisition and Analysis software (Olympus).

## Additional Information

**How to cite this article**: Mistrik, M. *et al*. Cells and Stripes: A novel quantitative photo-manipulation technique. *Sci. Rep.* **6**, 19567; doi: 10.1038/srep19567 (2016).

## Supplementary Material

Supplementary Information

## References

[b1] Kong X. . Comparative analysis of different laser systems to study cellular responses to DNA damage in mammalian cells. Nucleic acids research 37, e68, 10.1093/nar/gkp221 (2009). 19357094 PMC2685111

[b2] Dinant C. . Activation of multiple DNA repair pathways by sub-nuclear damage induction methods. Journal of cell science 120, 2731–2740, 10.1242/jcs.004523 (2007). 17646676

[b3] Ishikawa-Ankerhold H. C., Ankerhold R. & Drummen G. P. Advanced fluorescence microscopy techniques—FRAP, FLIP, FLAP, FRET and FLIM. Molecules (Basel, Switzerland) 17, 4047–4132, 10.3390/molecules17044047 (2012). PMC6268795 22469598

[b4] Britton S. . DNA damage triggers SAF-A and RNA biogenesis factors exclusion from chromatin coupled to R-loops removal. Nucleic acids research 42, 9047–9062, 10.1093/nar/gku601 (2014). 25030905 PMC4132723

[b5] Lukas C., Falck J., Bartkova J., Bartek J. & Lukas J. Distinct spatiotemporal dynamics of mammalian checkpoint regulators induced by DNA damage. Nature cell biology 5, 255–260, 10.1038/ncb945 (2003). 12598907

[b6] Galanty Y. . Mammalian SUMO E3-ligases PIAS1 and PIAS4 promote responses to DNA double-strand breaks. Nature 462, 935–939, 10.1038/nature08657 (2009). 20016603 PMC2904806

[b7] Bekker-Jensen S. . Spatial organization of the mammalian genome surveillance machinery in response to DNA strand breaks. The Journal of cell biology 173, 195–206, 10.1083/jcb.200510130 (2006). 16618811 PMC2063811

[b8] Bekker-Jensen S., Lukas C., Melander F., Bartek J. & Lukas J. Dynamic assembly and sustained retention of 53BP1 at the sites of DNA damage are controlled by Mdc1/NFBD1. The Journal of cell biology 170, 201–211, 10.1083/jcb.200503043 (2005). 16009723 PMC2171401

[b9] Jackson S. P. & Bartek J. The DNA-damage response in human biology and disease. Nature 461, 1071–1078, 10.1038/nature08467 (2009). 19847258 PMC2906700

[b10] Lukas C. . Mdc1 couples DNA double-strand break recognition by Nbs1 with its H2AX-dependent chromatin retention. The EMBO journal 23, 2674–2683, 10.1038/sj.emboj.7600269 (2004). 15201865 PMC449779

[b11] Lukas J., Lukas C. & Bartek J. More than just a focus: The chromatin response to DNA damage and its role in genome integrity maintenance. Nature cell biology 13, 1161–1169, 10.1038/ncb2344 (2011). 21968989

[b12] Stucki M. & Jackson S. P. gammaH2AX and MDC1: anchoring the DNA-damage-response machinery to broken chromosomes. DNA repair 5, 534–543, 10.1016/j.dnarep.2006.01.012 (2006). 16531125

[b13] Coster G. & Goldberg M. The cellular response to DNA damage: a focus on MDC1 and its interacting proteins. Nucleus (Austin, Tex.) 1, 166–178, 10.4161/nucl.1.2.11176 (2010). PMC3030693 21326949

[b14] Doil C. . RNF168 binds and amplifies ubiquitin conjugates on damaged chromosomes to allow accumulation of repair proteins. Cell 136, 435–446, 10.1016/j.cell.2008.12.041 (2009). 19203579

[b15] Panier S. & Boulton S. J. Double-strand break repair: 53BP1 comes into focus. Nature reviews. Molecular cell biology 15, 7–18, 10.1038/nrm3719 (2014). 24326623

[b16] Kim H. & D’Andrea A. D. Regulation of DNA cross-link repair by the Fanconi anemia/BRCA pathway. Genes & development 26, 1393–1408, 10.1101/gad.195248.112 (2012). 22751496 PMC3403008

[b17] Xu G. . REV7 counteracts DNA double-strand break resection and affects PARP inhibition. Nature 521, 541–544, 10.1038/nature14328 (2015). 25799992 PMC4671316

[b18] Toledo L. I. . ATR prohibits replication catastrophe by preventing global exhaustion of RPA. Cell 155, 1088–1103, 10.1016/j.cell.2013.10.043 (2013). 24267891

[b19] Epe B. DNA damage spectra induced by photosensitization. Photochemical & photobiological sciences: Official journal of the European Photochemistry Association and the European Society for Photobiology 11, 98–106, 10.1039/c1pp05190c (2012). 21901212

[b20] Miller K. M. . Human HDAC1 and HDAC2 function in the DNA-damage response to promote DNA nonhomologous end-joining. Nature structural & molecular biology 17, 1144–1151, 10.1038/nsmb.1899 (2010). PMC3018776 20802485

[b21] Fujii Y. . Comparison of the bromodeoxyuridine-mediated sensitization effects between low-LET and high-LET ionizing radiation on DNA double-strand breaks. Oncol Rep 29, 2133–2139, 10.3892/or.2013.2354 (2013). 23525528

[b22] Dinant C. . Enhanced chromatin dynamics by FACT promotes transcriptional restart after UV-induced DNA damage. Molecular cell 51, 469–479, 10.1016/j.molcel.2013.08.007 (2013). 23973375

[b23] Chirnomas D. . Chemosensitization to cisplatin by inhibitors of the Fanconi anemia/BRCA pathway. Molecular cancer therapeutics 5, 952–961, 10.1158/1535-7163.mct-05-0493 (2006). 16648566

[b24] Reaper P. M. . Selective killing of ATM- or p53-deficient cancer cells through inhibition of ATR. Nature chemical biology 7, 428–430, 10.1038/nchembio.573 (2011). 21490603

[b25] King C. . Characterization and preclinical development of LY2603618: a selective and potent Chk1 inhibitor. Investigational new drugs 32, 213–226, 10.1007/s10637-013-0036-7 (2014). 24114124

[b26] Wang G., Bhoopalan V., Wang D., Wang L. & Xu X. The effect of caffeine on cisplatin-induced apoptosis of lung cancer cells. Experimental hematology & oncology 4, 5, 10.1186/2162-3619-4-5 (2015). 25937999 PMC4417201

[b27] Gurung R. L., Lim H. K., Venkatesan S., Lee P. S. & Hande M. P. Targeting DNA-PKcs and telomerase in brain tumour cells. Molecular cancer 13, 232, 10.1186/1476-4598-13-232 (2014). 25307264 PMC4213508

[b28] Hopkins T. A. . Mechanistic Dissection of PARP1 Trapping and the Impact on *In Vivo* Tolerability and Efficacy of PARP Inhibitors. Molecular cancer research : MCR 13, 1465–1477, 10.1158/1541-7786.mcr-15-0191-t (2015). 26217019

[b29] Hickson I. . Identification and characterization of a novel and specific inhibitor of the ataxia-telangiectasia mutated kinase ATM. Cancer research 64, 9152–9159, 10.1158/0008-5472.can-04-2727 (2004). 15604286

[b30] Lund-Andersen C., Patzke S., Nahse-Kumpf V. & Syljuasen R. G. PLK1-inhibition can cause radiosensitization or radioresistance dependent on the treatment schedule. Radiotherapy and oncology: journal of the European Society for Therapeutic Radiology and Oncology 110, 355–361, 10.1016/j.radonc.2013.12.014 (2014). 24502970

[b31] Liu Y. . Targeting aurora kinases limits tumour growth through DNA damage-mediated senescence and blockade of NF-kappaB impairs this drug-induced senescence. EMBO molecular medicine 5, 149–166, 10.1002/emmm.201201378 (2013). 23180582 PMC3569660

[b32] Jekimovs C. . Chemotherapeutic compounds targeting the DNA double-strand break repair pathways: the good, the bad, and the promising. Frontiers in oncology 4, 86, 10.3389/fonc.2014.00086 (2014). 24795863 PMC4001069

[b33] Muraki K., Han L., Miller D. & Murnane J. P. Processing by MRE11 is involved in the sensitivity of subtelomeric regions to DNA double-strand breaks. Nucleic acids research 43, 7911–7930, 10.1093/nar/gkv714 (2015). 26209132 PMC4652756

