# Supplementary material for: Cells and Stripes: A novel quantitative photo-manipulation technique
Source: Sci Rep. 2016 Jan 18;6:19567. doi: 10.1038/srep19567 (PMC4726120; doi:10.1038/srep19567)
Supplement: Supplementary Information [file srep19567-s1.pdf]

Supplementary Figures

Cells and Stripes: A novel quantitative photo-manipulation technique

Martin Mistrik, Eva Vesela, Tomas Furst, Hana Hanzlikova, Ivo Frydrych, Jan Gursky, Dusana Majera and Jiri Bartek

Supplementary Fig. S1

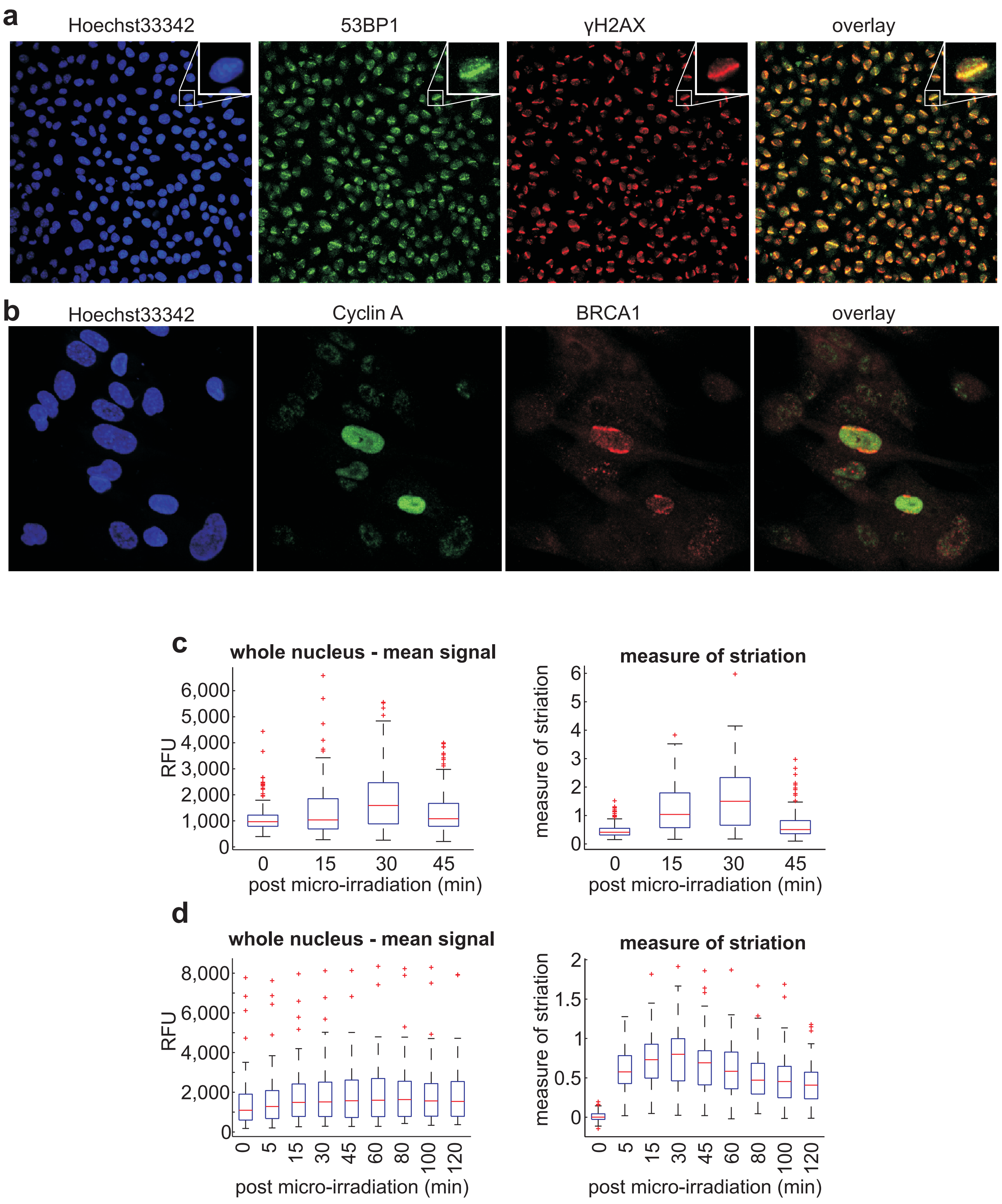

Supplementary Figure S1: | Method application for immunofluorescence (IF) and comparison of signal quantifications. **(a)** IF detection of endogenous 53BP1 and γH2AX proteins at damaged sites in a mouse McCoy cell line. Cells were BrdU pre-sensitized, irradiated by a 355nm laser (32 lines/field, 2 iterations) and fixed 30 min after irradiation. **(b)** Example of combined IF staining of endogenous CyclinA (cell-cycle stage marker) and BRCA1 protein (S-phase related DNA damage response/repair protein) pointing at rare event applications of the method (all cells in the field were irradiated, but only CyclinA positive show the stripe response). Experiment was performed in MRC-5 cells, BrdU pre-sensitized, irradiated by a 355nm laser (32 lines/field, 5 iterations) and fixed 30 min after irradiation. **(c)** Comparison of different types of signal quantification of phosphorylated histone H2AX (γH2AX). MRC-5 cells were BrdU pre-sensitized, irradiated by a 355nm laser (32 lines/field, 5 iterations) and fixed at the indicated time points. Signal was plotted either as mean signal per nucleus (left panel) or measure of striation (right panel). **(d)** Comparison of different types of signal quantification for MDC1-GFP protein which translocates into the sites of damage. U-2OS-MDC1-GFP cell line was BrdU pre-sensitized, irradiated by a 355nm laser (32 lines/field, 1 iteration) and fixed at the indicated time points. Signal was plotted either as mean signal per nucleus (left panel) or measure of striation (right panel).

Supplementary Fig. 2

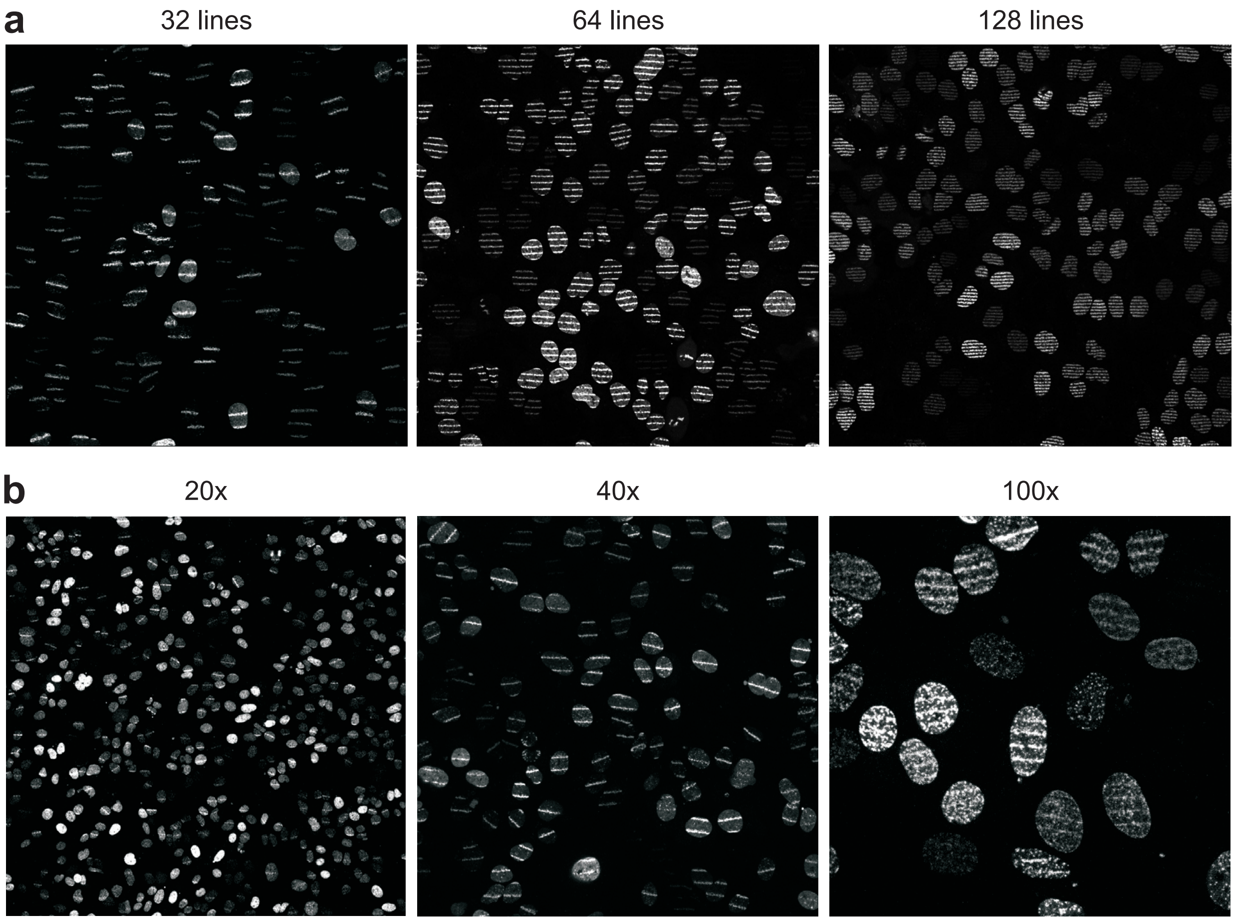

c

magnification	immersion	stripes formation	objective type
20x	none	yes	Zeiss, Pln-Apo, 0.8, DICII
20x	water	no	Zeiss, Pln-Apo, 1W, DICIII
20x	water	no	Zeiss, N-ACP, 0.5W
40x	oil	yes	Zeiss, EC Pln 1.30 DICII
40x	water	yes	Zeiss, C-Apo, 1.2 DICIII
63x	oil	yes	Zeiss, Alpha Pln Apo 63x/1.40
100x	oil	yes	Zeiss, Pln Apo100x/1.46

Supplementary Figure S2: | Effect of used objectives and LSM settings on the striation pattern. (a) The gauge of the striation pattern (amount of lines per field) set as the scanning resolution of LSM system. (b) Effect of the objective magnification at fixed scanning resolution (32 lines/field) on the amount and quality of stripes per nucleus. U-2-OS-MDC1-GFP cell line was BrdU pre-sensitized and irradiated by a 355nm laser (60 iteration for 20x, 10 iterations for 40x and 60 iterations for 100x objectives). Striation pattern was acquired 30 min after irradiation. (c) List of tested objectives on Zeiss LSM780 system with respect to their ability to form stripes in BrdU pre-sensitized U-2-OS-MDC1-GFP cells using a 355nm laser.

Supplementary Fig. S3

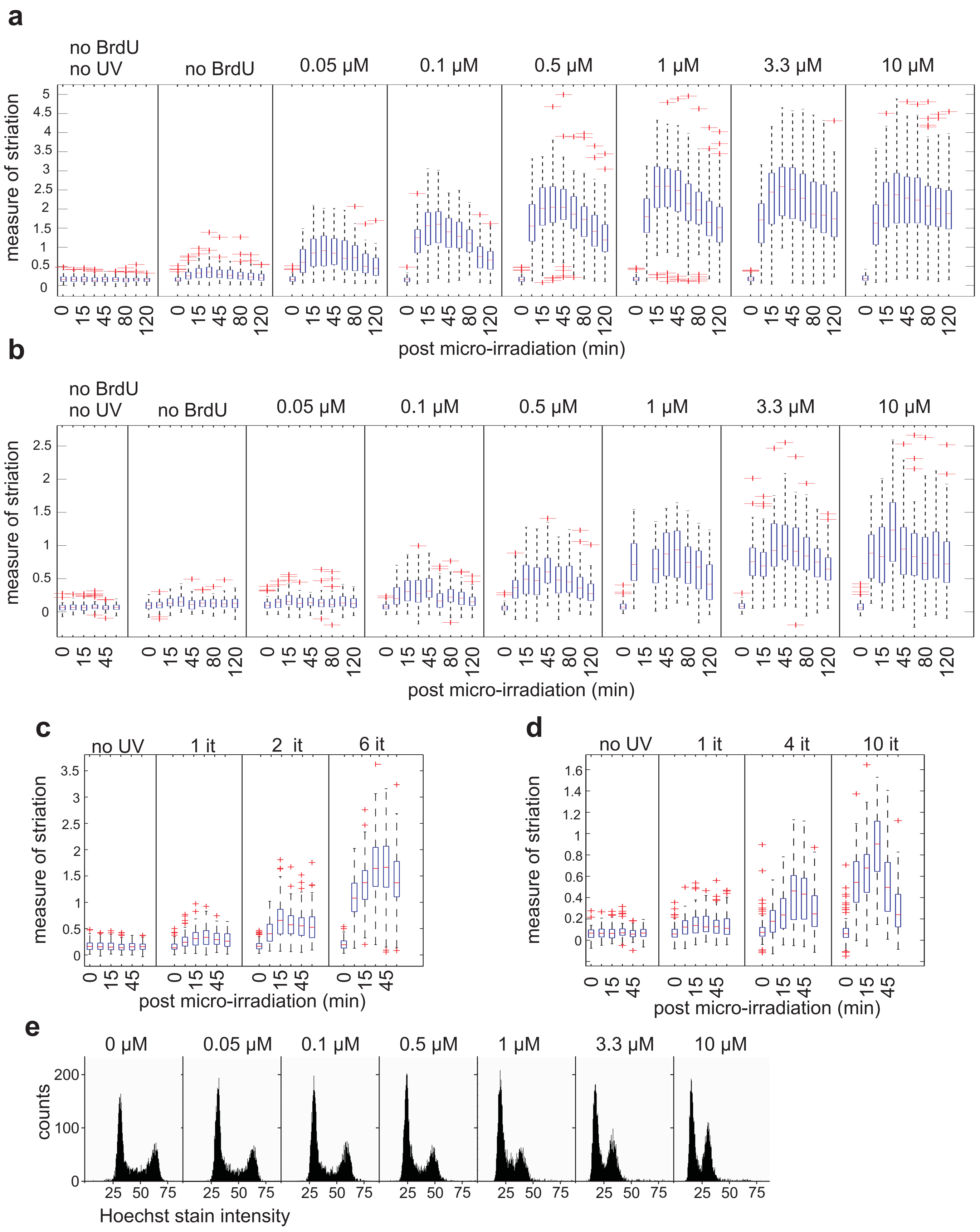

Supplementary Figure S3: | Effect of BrdU presensitization on striation pattern evolution and the cell cycle. **(a)** Measure of striation after constant laser power applied to cells pre-incubated for 24h with indicated concentrations of BrdU, a 355nm laser (32 lines/field, 1 iteration) in U-2-OS-MDC1-GFP cells. **(b)** Measure of striation after constant laser power applied to cells pre-incubated for 24h with indicated concentrations of BrdU, a 405nm laser (32 lines/field, 1iteration) in the U-2-OS-MDC1-GFP cell line. **(c)** Measure of striation reflecting various irradiation doses of a 355nm laser in non-presensitized U-2-OS-MDC1-GFP cells (32 lines/field). Irradiation dose was manipulated by the number of iterations at 100% laser power. **(d)** Measure of striation reflecting various irradiation doses of a 405nm laser in non-presensitized U-2-OS-MDC1-GFP cells (32 lines/field). Irradiation dose was manipulated by the number of iterations at 68% laser power. **(e)** Effect of selected concentrations of BrdU after 24-h incubation on cell cycle progression in U-2-OS-MDC1-GFP cells.
